# SARS-CoV-2 infects cells after viral entry via clathrin-mediated endocytosis

**DOI:** 10.1016/j.jbc.2021.100306

**Published:** 2021-01-19

**Authors:** Armin Bayati, Rahul Kumar, Vincent Francis, Peter S. McPherson

**Affiliations:** Department of Neurology and Neurosurgery, Montreal Neurological Institute, McGill University, Montreal, Quebec, Canada

**Keywords:** clathrin, COVID-19, dynamin, endocytosis, infection, SARS-CoV-2, virus entry, ACE2, angiotensin-converting enzyme 2, CHC, clathrin heavy chain, CQ, chloroquine, MERS-CoV, Middle-East respiratory syndrome coronavirus, SARS-CoV, severe acute respiratory syndrome coronavirus, SARS-CoV-2, severe acute respiratory syndrome coronavirus 2, Tf, transferrin, TfR, Tf receptor

## Abstract

Severe acute respiratory syndrome coronavirus 2 (SARS-CoV-2) is the causative agent of COVID-19, so understanding its biology and infection mechanisms is critical to facing this major medical challenge. SARS-CoV-2 is known to use its spike glycoprotein to interact with the cell surface as a first step in the infection process. As for other coronaviruses, it is likely that SARS-CoV-2 next undergoes endocytosis, but whether or not this is required for infectivity and the precise endocytic mechanism used are unknown. Using purified spike glycoprotein and lentivirus pseudotyped with spike glycoprotein, a common model of SARS-CoV-2 infectivity, we now demonstrate that after engagement with the plasma membrane, SARS-CoV-2 undergoes rapid, clathrin-mediated endocytosis. This suggests that transfer of viral RNA to the cell cytosol occurs from the lumen of the endosomal system. Importantly, we further demonstrate that knockdown of clathrin heavy chain, which blocks clathrin-mediated endocytosis, reduces viral infectivity. These discoveries reveal that SARS-CoV-2 uses clathrin-mediated endocytosis to gain access into cells and suggests that this process is a key aspect of virus infectivity.

The internal membrane system represents a major evolutionary advance associated with the emergence of the eukaryotic cell. Its dynamics are integral to basic cellular function and underlie much of the novel capability of the cell, including the almost infinite range of physiological responsiveness required for the complexity of eukaryotic organisms. Unfortunately, viruses subvert this complexity to gain access into cells and to propagate in a manner that allows them to limit immune surveillance. Antiviral strategies targeting the earliest steps of infection, such as cellular entry, are appealing, as interference through a common early step can provide robust efficacy. Thus, understanding the mechanisms of viral cellular entry is crucial.

Since the beginning of the 21st century, 3 coronaviruses have crossed the species barrier to cause deadly types of pneumonia in humans: Middle-East respiratory syndrome coronavirus (MERS-CoV) (1), severe acute respiratory syndrome coronavirus (SARS-CoV) (2, 3), and SARS-CoV-2 (4, 5), the causative agent of COVID-19. All originated in bats, but zoonotic transmission involved intermediate hosts; dromedary camels (MERS-CoV), civets (SARS-CoV), and unknown (SARS-CoV-2). The presence of numerous coronaviruses in bats suggests that zoonotic transmission to humans will continue (6), and new pandemic potential viruses continue to emerge (7).

SARS-CoV-2 is a single-stranded RNA virus in which a lipid membrane surrounds the RNA and nucleocapsid proteins. Other viral structural proteins associate with the viral membrane including the spike protein, a transmembrane glycoprotein that forms homotrimers (6). The spike glycoprotein is composed of S1 and S2 subdomains (8). The S1 subdomain encodes the receptor binding domain and is responsible for binding to host cells, whereas the S2 subdomain encodes the transmembrane portion of the spike protein and is responsible for fusion of the viral membrane with cellular membranes. The receptor for the spike glycoprotein is angiotensin-converting enzyme 2 (ACE2) (9, 10, 11, 12) although recent studies indicate that neuropilin-1 is also a host factor for SARS-CoV-2 and facilitates infectivity (13, 14, 15). ACE2 is a transmembrane metallopeptidase localized primarily on the plasma membrane of many cell types with abundant expression in lung alveolar epithelial cells (16). The receptor binding domain of the spike protein binds ACE2 with low nM affinity, allowing the virus to stably associate with the plasma membrane (12). Cleavage of the spike glycoprotein between the S1 and S2 domains, mediated by the type II transmembrane serine protease TMPRSS2 (9), and perhaps by furin, activates the S2 subdomain. The S2 subdomain then mediates the fusion of the viral membrane and the cellular membrane in a process that is a molecular mimic of SNARE-mediated fusion of cellular membranes (12, 16, 17). This creates a pore allowing the RNA and RNA-associated nucleocapsid proteins within the lumen of the virus to gain access to the cellular cytosol, triggering infection.

For SARS-CoV-2, it remains unknown where the fusion of the viral and cellular membranes occurs. One possibility is that fusion occurs at the cell plasma membrane (18). In such a scenario, the virus does not directly enter the cell, but the viral RNA that drives infection enters the cytosol through a fusion pore in the plasma membrane. Alternatively, SARS-CoV-2 may undergo endocytosis with the entire viral particle rapidly entering the cell. In this scenario, the viral membrane would fuse with the luminal face of the endosomal membrane, allowing for RNA transfer to the cytosol. In fact, it appears that most human coronaviruses are engulfed/endocytosed before they infect the cell with their genetic material. This includes human (H)CoV-229E (19, 20) HCoV-OC43 (21), HCoV-NL63 (22, 23), HCoV-HKU1 (24), MERS-CoV (25, 26), and SARS-CoV (27, 28). And yet, the endocytic mechanisms used by these coronaviruses remain unclear as they have been shown to use (1) clathrin-dependent, (2) caveolae-dependent/clathrin-independent, and (3) caveolae- and clathrin-independent pathways. In fact, the virus most similar to SARS-CoV-2 is SARS-CoV, and there are conflicting results regarding its endocytic entry; one study indicating that the virus uses clathrin-mediated endocytosis (26) and a second stating the opposite that viral entry before infectivity uses a clathrin-independent process (27). Here we demonstrate rapid, clathrin-mediated endocytosis of SARS-CoV-2 and provide evidence that this process is critical for infectivity.

## Experimental procedures

### Cell lines

HEK-293T and A549 cell lines were obtained from the American Type Culture Collection (CRL-1573, CCL-185). The adherent Vero-SF-ACF cell line is from the American Type Culture Collection (CCL-81.5) (29). The HEK-293T-ACE2 stable cell line was a gift from Dr Jesse D. Bloom (30).

### Cell culture

All cells were cultured in Dulbecco's modified Eagle's medium (DMEM) high-glucose (GE Healthcare cat# SH30081.01) containing 10% bovine calf serum (GE Healthcare cat# SH30072.03), 2-mM L-glutamate (Wisent cat# 609065), 100 IU penicillin, and 100 μg/ml streptomycin (Wisent cat# 450201). Cell lines were checked monthly for *Mycoplasma* contamination using the *Mycoplasma* detection kit (biotool cat# B39038).

### Immunoblot

Cells were collected in Hepes lysis buffer (20-mM Hepes, 150-mM sodium chloride, 1% Triton X-100, pH 7.4) supplemented with protease inhibitors. Cells in the lysis buffer were gently rocked for 30 min (4 °C). Lysates were spun at 238,700g for 15 min at 4 °C and equal protein aliquots of the supernatants were analyzed by SDS-PAGE and immunoblot. Lysates were run on large 5 to 16% gradient polyacrylamide gels and transferred to nitrocellulose membranes. Proteins on the blots were visualized by Ponceau staining. Blots were then blocked with 5% milk, and antibodies were incubated O/N at 4 °C with 5% bovine serum albumin in tris-buffered saline with 0.1% Tween 20. The peroxidase conjugated secondary antibody was incubated in a 1:5000 dilution in tris-buffered saline with 0.1% Tween 20 with 5% milk for 1 h at room temperature (RT) followed by washes.

### Antibodies

SARS-CoV-2 spike protein antibody is from GeneTex (GTX632604). ACE2 antibody is from GeneTex (GTX01160). 6x-His Tag antibody is from Thermo Fisher Scientific (MA1-21315-D550). GAPDH antibody is from OriGene (TA802519). HSC70 antibody is from Enzo (ADI-SPA-815-F). Clathrin heavy chain (CHC) antibody is from Cell Signaling Technology (4796S). Conjugated transferrin (Tf) antibody is from Thermo Fisher Scientific (T23366). Alexa Fluor 488, 568, and 647 conjugated secondary antibodies are from Invitrogen.

### Confocal microscopy

Cells grown on poly-L-lysine–coated coverslips were fixed in 4% paraformaldehyde for 10 min and then washed 3 times with PBS. Cells were then permeabilized in 0.2% Triton X-100 in PBS and blocked with 5% bovine serum albumin in PBS for 1 h. The coverslips were incubated in a wet chamber with diluted antibody in a blocking buffer overnight at 4 °C. The following day, cells were washed 3 times and incubated with corresponding Alexa Fluorophore diluted in the blocking buffer for 1 h at RT. Cells were again washed 3 times with a blocking buffer and once with PBS. Nuclei were stained using 4′,6-diamidino-2-phenylindole (DAPI) (1 μg/ml diluted in a blocking buffer) for 10 min. Finally, coverslips were mounted on a slide using mounting media (DAKO, Cat# S3023). Imaging was performed using a Leica TCS SP8 confocal microscope, Zeiss LSM-880 confocal microscope, and Opera Phoenix High-Content Screening microscope.

### Endocytosis assay using purified spike protein

Cells were incubated at 37 °C with serum-free media (DMEM) for 3 h to enhance ACE2 receptor expression. Before the addition of spike protein, cells were cooled to 4 °C by being placed on ice. Spike protein was added to each well (3 μg per 200 μl of media) and incubated on ice for 30 min (to allow ligand attachment to the cell surface). Subsequently, cells were incubated at 37 °C (to allow internalization) for indicated time points (Tf was also added in some experiments in the same manner). Before fixation, cells were acid washed (washing off extracellular spike protein) or PBS washed for 1 min and rinsed with acid or PBS. Cells were then washed 3 times with PBS followed by fixation for 10 min at 4 °C.

### siRNA-mediated knockdown of CHC

Various cell types at 60% confluency were transfected with siRNA targeted against CHC (Dharmacon; SMARTpool: ON-TARGETplus; L-004001–01–0010) or control siRNA (Dharmacon; ON-TARGETplus CONTROL) using the jetPRIME reagent. On day 3, cells were processed for immunoblot to investigate the effect of siRNA, and in parallel, cells were infected with pseudovirus or used for spike endocytosis assays.

### Purified SARS-Cov-2 spike and pseudovirus production

Purified SARS-CoV-2 spike protein prefusion-stabilized ectodomain (C-term His tag, with furin cleavage site removed, trimerization stabilized) was produced by LakePharma (#46328). Lentivirus pseudotyped with SARS-CoV-2 spike protein was supplied by Creative Diagnostics (catalog number COV-PS02).

### Treatments with chemical inhibitors

Cells were washed five times with serum-free media (DMEM) to remove any traces of serum. Furthermore, cells were incubated with Dynasore (80 μM; Abcam, Ab120192) or Pitstop 2 (15 μM; Abcam, ab120687) in serum-free media for 30 or 20 min, respectively. DMSO was used as a control for Dynasore and Pitstop 2 negative control (Abcam, ab120688). After incubation, drug-containing media was replaced with spike protein (3 μg per 200 μl of media) and further incubated for 5 min to allow internalization. Finally, cells were processed for immunofluorescence labeling.

### Pseudovirus SARS-Cov-2 infection

Cells were transfected with control siRNA or siRNA targeted against CHC (Dharmacon) using jetPRIME. Cells were split on day 2, and 10,000 cells were seeded in each well (96-well plate; CellCarrier-96 Ultra Microplates, PerkinElmer). At 12 to 14 h after cell seeding, cells were incubated with concentrated pseudovirus SARS-Cov-2 for 12 h. After this incubation, pseudovirus containing media was replaced with regular DMEM media and incubated further for 48 h. Cells were fixed with 4% paraformaldehyde for 10 min and stained with DAPI.

### Quantification

Purified spike protein and Tf (internalized or on the plasma membrane): ImageJ was used to measure the fluorescence intensity. The DAPI channel was used to count the number of cells per image. This was performed by calculating the maxima using the “Find Maxima” function. The threshold was set at 150. This automated the way in which to count the cells. Then, spike protein or Tf fluorescence corresponding to the cell count was calculated using the “measure” function. Pearson Correlation Coefficient: Using ImageJ Coloc2 plugin, colocalization was calculated after background subtraction. The colocalization was calculated for 144 × 144 μm. Fluorescence was then divided by cell count to calculate the fluorescence per cell in each condition. Infection of cells using pseudovirus SARS-Cov-2: GFP-expressing cells in CellCarrier-96 Ultra Microplate were imaged using Opera Phenix HCS (40× objective). The total number of GFP expressing cells in each condition was normalized to the total number of cells in the respective wells.

### Statistics

Graphs were prepared using GraphPad Prism 6 software. For all data, comparisons were made using Student's *t* test or one-way ANOVA. All data are shown as the mean ± SEM with *p* < 0.05 considered statistically significant.

## Results

### SARS-CoV-2 spike glycoprotein is rapidly endocytosed in an ACE2-dependent manner

**Figure 1:**
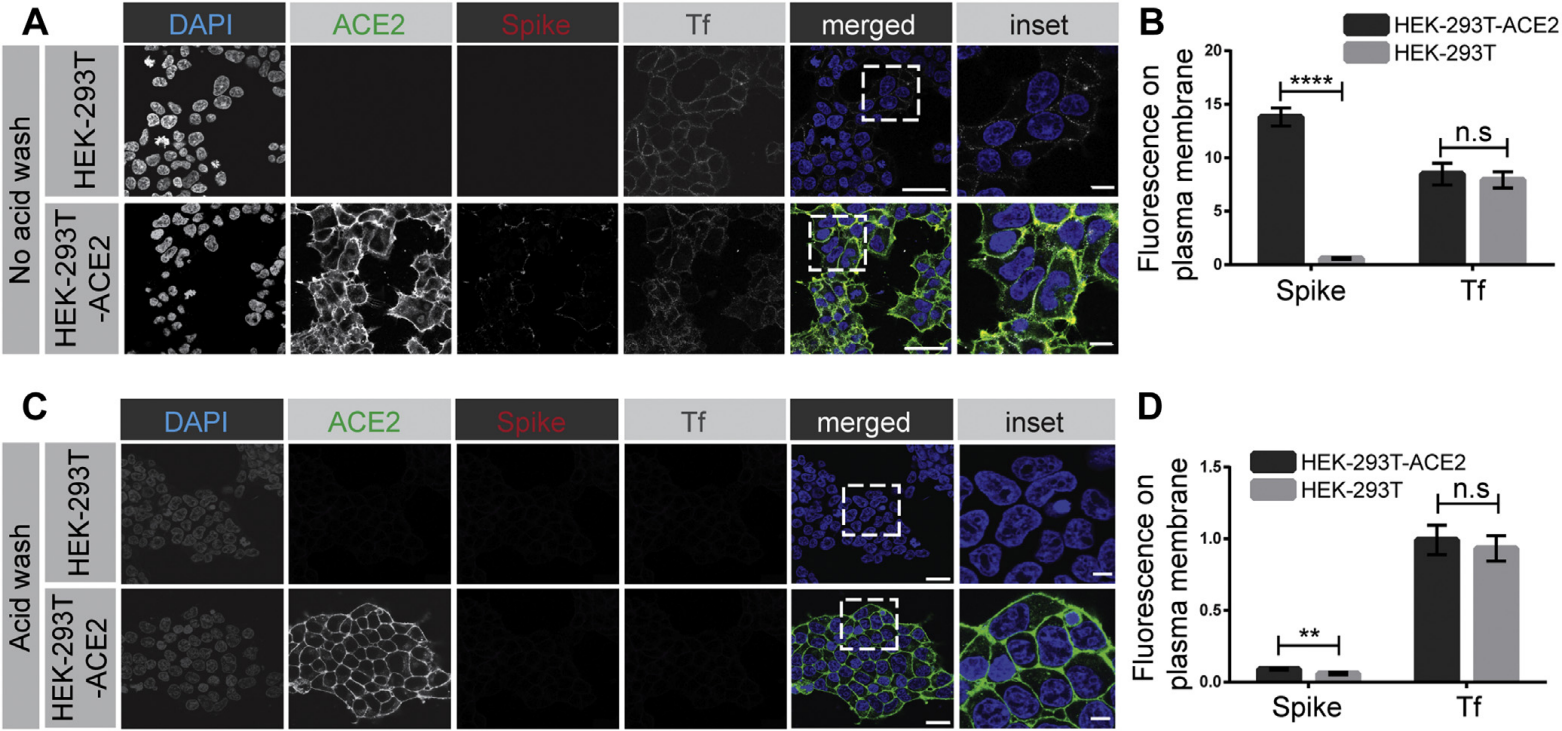
**SARS-CoV-2 spike protein binds to the surface of HEK-293T cells expressing ACE2**. *A*, HEK-293T cells, WT (*top row* of images), or stably expressing ACE2 (*bottom row* of images) were incubated with purified, His6-tagged spike protein and with Alexa 647–labeled transferrin (Tf) for 30 min at 4 °C. After PBS wash, the cells were fixed and stained with DAPI to reveal nuclei, with an antibody recognizing the expressed ACE2, and with an antibody recognizing the His6 epitope tag of the spike protein. Scale bars = 40 μm for the low-magnification images and 10 μm for the higher magnification *inset* of the merged images. *B*, quantification of experiments as in (*A*). The bar graph represents fluorescence on the plasma membrane for spike protein and Tf, from three independent experiments, mean ± SEM; unpaired *t* test; ∗∗∗∗*p* < 0.0001, n.s, not significant. *C*, experiments performed as in (*A*) except that the HEK-293T cells were briefly acid-washed before fixation. Scale bars = 40 μm for the low magnification images and 10 μm for the higher magnification *inset* of the composite. *D*, quantification of experiments as in (*C*). The bar graph represents fluorescence on the plasma membrane for spike protein and Tf, from three independent experiments, mean ± SEM; unpaired *t* test; ∗∗∗∗*p* < 0.0001, ∗∗*p* < 0.01, n.s, not significant. ACE2, angiotensin-converting enzyme 2; SARS-CoV-2, severe acute respiratory syndrome coronavirus 2.

The spike glycoprotein is critical for binding ACE2 and allowing for SARS-CoV-2 infectivity (6, 8, 9, 10, 11, 12, 16, 17). WT HEK-293T cells or HEK-293T cells stably expressing ACE2 (30) were incubated for 30 min on ice with purified spike protein containing a His6 tag. After a PBS wash, spike protein binds to the plasma membrane of cells expressing ACE2 but not to the control HEK-293T cells (Fig. 1, *A* and *B*). In contrast, Tf, which binds to the Tf receptor (TfR), is detected on the surface of the cells independent of their ACE2 status (Fig. 1, *A* and *B*). After a brief acid wash, both spike protein and Tf are stripped from the surface of cells (Fig. 1, *C* and *D*). Thus, HEK-293T cells lacking or expressing ACE2 are a functional model system for examining potential SARS-CoV-2 endocytosis.

**Figure 2:**
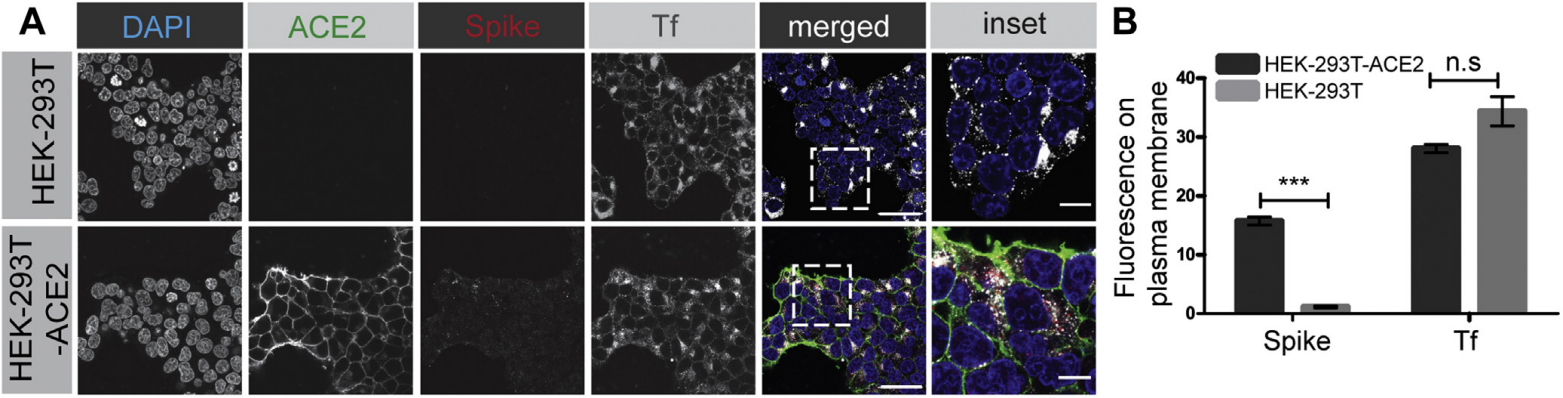
**SARS-CoV-2 spike protein enters cells in an ACE2-dependent manner**. *A*, HEK-293T cells, WT (*top row* of images), or stably expressing ACE2 (*bottom row* of images) were incubated with purified, His6-tagged spike protein and with Alexa 647–labeled transferrin (Tf) for 30 min at 4 °C. The cells were then transferred to 37 °C for 30 min. The cells were returned to ice and after acid wash, were fixed and stained with DAPI to reveal nuclei, with an antibody recognizing the expressed ACE2, and with an antibody recognizing the His6 epitope tag of the spike protein. Scale bars = 40 μm for the low-magnification images and 10 μm for the higher magnification *inset* of the composite. *B*, quantification of experiment as in (*A*). The bar graph represents fluorescence on the plasma membrane for spike protein and Tf, from three independent experiments, mean ± SEM; unpaired *t* test; ∗∗∗*p* < 0.001, n.s, not significant. ACE2, angiotensin-converting enzyme 2; SARS-CoV-2, severe acute respiratory syndrome coronavirus 2.

We first tested for endocytosis using purified spike glycoprotein. Although a reductionist approach, it is unlikely that receptor binding and initial membrane trafficking depends on whether or not the spike protein is on a viral particle. For example, epidermal growth factor and insulin both undergo similar cell surface binding and clathrin-mediated endocytosis whether they are purified proteins or attached to magnetic beads (31). We thus added purified spike protein to HEK-293T cells, WT or expressing ACE2. After 30 min on ice, the cells were transferred to 37 °C for an additional 30 min. After this treatment, cells were washed briefly with acid to strip off any surface-bound spike protein. Spike protein was internalized into the cells (as revealed by its resistance to acid wash) in an ACE2-dependent manner (Fig. 2, *A* and *B*). In contrast, Tf was internalized independent of ACE2 (Fig. 2, *A* and *B*).

**Figure 3:**
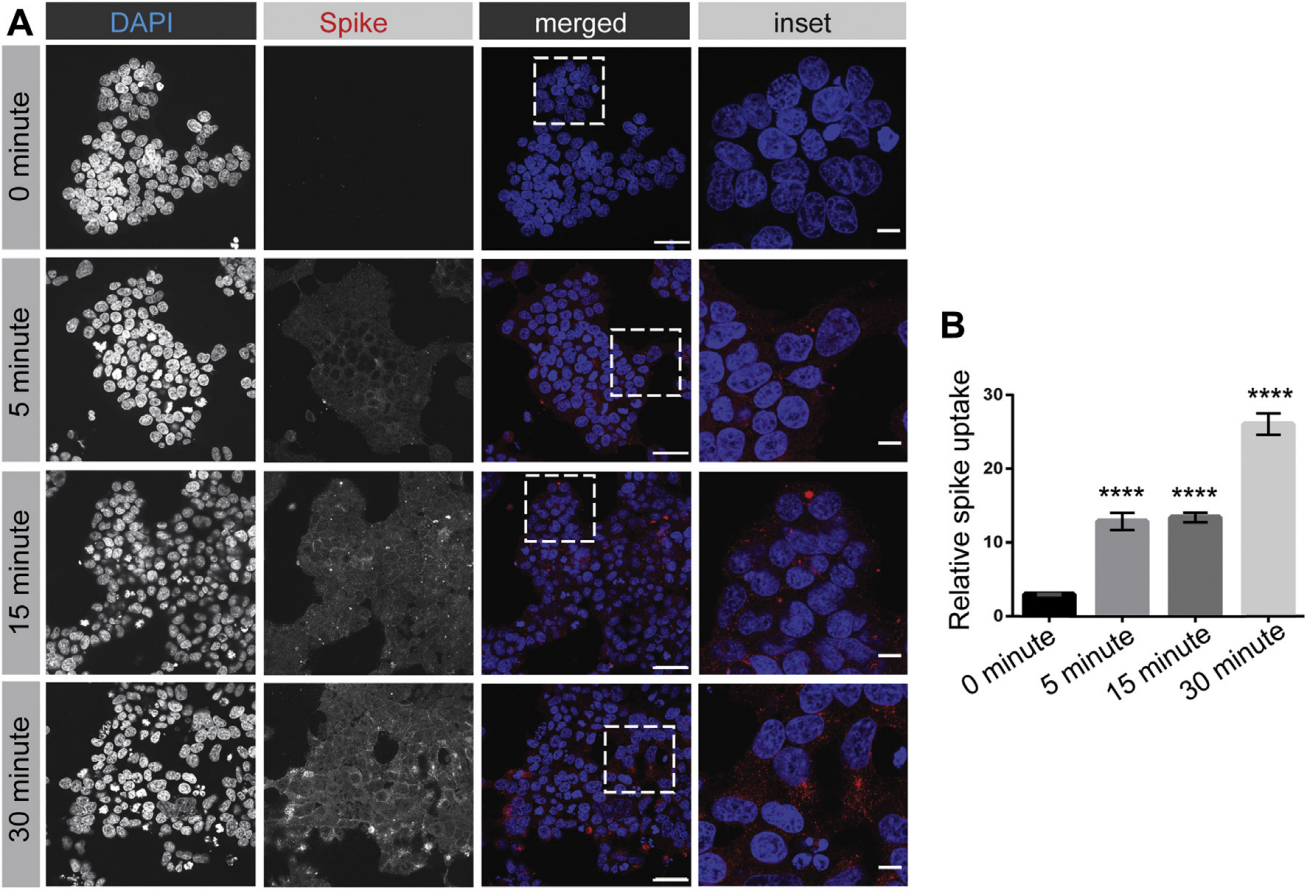
**Time course of SARS-CoV-2 spike protein entry into cells**. *A*, HEK-293T cells stably expressing ACE2 were incubated with purified, His6-tagged spike protein for 30 min at 4 °C. The cells were then transferred to 37 °C for the indicated time periods before being returned to ice. After acid wash, cells were fixed and stained with DAPI to reveal nuclei and with antibody selectively recognizing the His6 epitope tag of the spike protein. Scale bars = 40 μm for the low-magnification images and 10 μm for the higher magnification *insets* on the right. *B*, quantification of experiments as in (*A*), from three independent experiments, mean ± SEM; one-way ANOVA; ∗∗∗∗*p* < 0.0001. ACE2, angiotensin-converting enzyme 2; SARS-CoV-2, severe acute respiratory syndrome coronavirus 2.

We next examined the time course of internalization. Spike protein is internalized into cells rapidly and is detected in cells within 5 min (Fig. 3, *A* and *B*), a hallmark of endocytosis. The amount of spike protein in cells continues to increase for up to 30 min (Fig. 3, *A* and *B*). Thus, SARS-CoV-2 spike protein enters cells via endocytosis.

### SARS-CoV-2 spike protein is internalized by clathrin-mediated endocytosis

**Figure 4:**
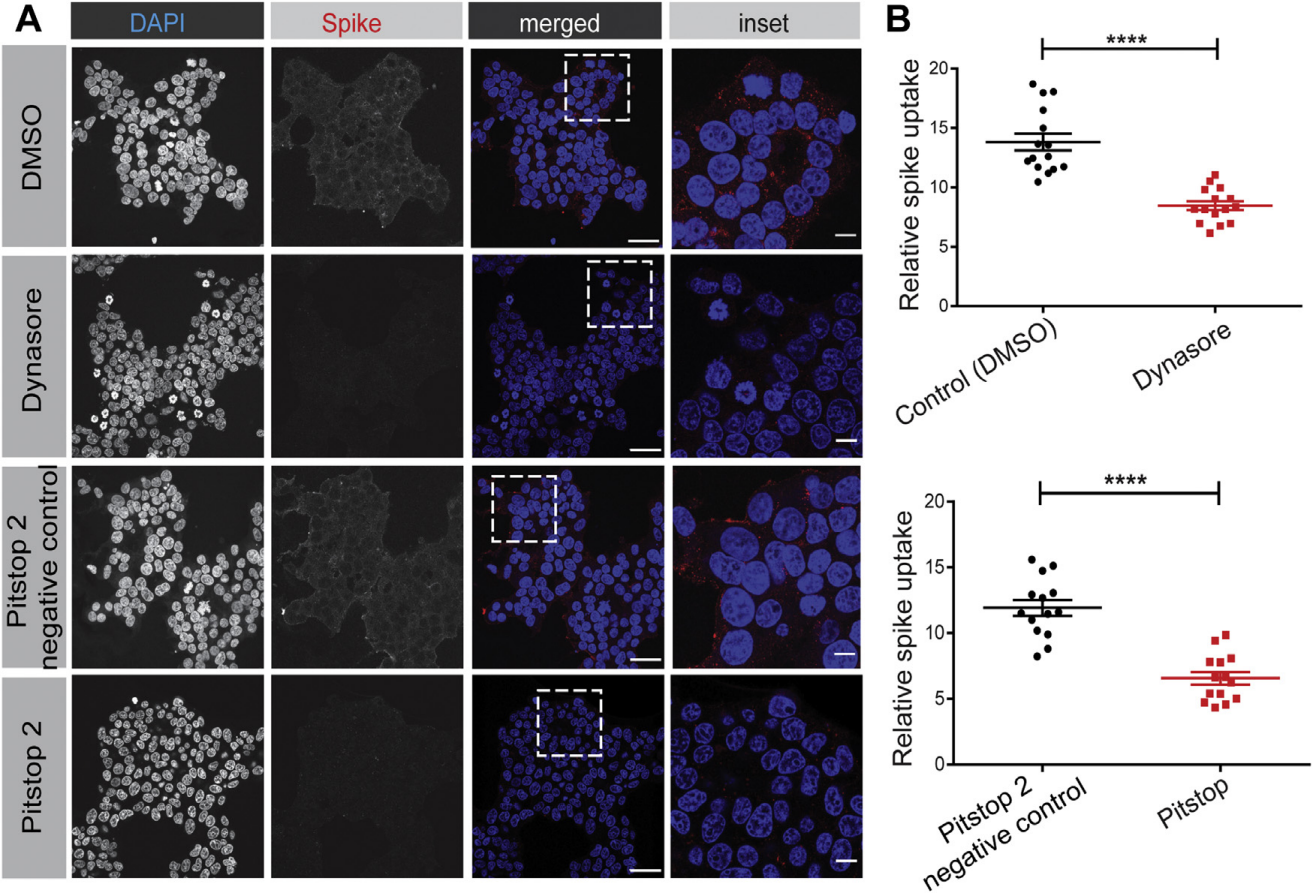
**SARS-CoV-2 spike protein endocytosis is blocked by chemical inhibitors of clathrin-mediated endocytosis**. *A*, HEK-293 cells stably expressing ACE2 were incubated with purified, His6-tagged spike protein for 30 min at 4 °C. The cells were then transferred to 37 °C for 30 min before being returned to ice. After acid wash, cells were fixed and stained with DAPI to reveal nuclei and with antibody selectively recognizing the His6 epitope tag of the spike protein. Inhibitors of clathrin-mediated endocytosis or their controls, as indicated, were added to the cells 30 min before the addition of spike protein. Scale bars = 40 μm for the low-magnification images and 10 μm for the higher magnification *insets* on the right. *B*, quantification of experiments performed as in (*A*). n = 15 for Dynasore and n = 14 for Pitstop 2 from three independent experiments, mean ± SEM; unpaired *t* test; ∗∗∗∗*p* < 0.0001. ACE2, angiotensin-converting enzyme 2; SARS-CoV-2, severe acute respiratory syndrome coronavirus 2.

There are conflicting reports about endocytosis of coronaviruses in general and SARS-CoV specifically (27, 28). The endocytic mechanisms of SARS-CoV-2 have yet to be examined. Clathrin-mediated endocytosis is a major mechanism for cellular internalization. We thus used HEK-293T cells stably expressing ACE2 to examine the internalization of SARS-CoV-2 in the presence of two drugs that are known to block clathrin-mediated endocytosis, Dynasore and Pitstop 2 (32, 33). Dynasore blocks the GTPase dynamin that drives the fission of clathrin-coated pits from the plasma membrane and it disrupts internalization of the TfR and low-density lipoprotein receptor, among other clathrin-dependent targets (32). Pitstop 2 prevents CHC from interacting with adaptor proteins required for clathrin-coated pit formation and blocks the uptake of TfR and epidermal growth factor receptor (33). Both drugs reduce the endocytosis of SARS-CoV-2 spike protein, strongly suggesting it is internalized by clathrin-mediated endocytosis (Fig. 4, *A* and *B*). Both Dynasore and Pitstop 2 have been criticized for having off-target effects (34, 35). However, at the concentrations and cell systems used here, while both drugs block the uptake of Tf, there is no influence of either drug on the uptake of dextran, which is internalized in a clathrin-independent manner (Fig. S1, *A* and *B*).

**Figure 5:**
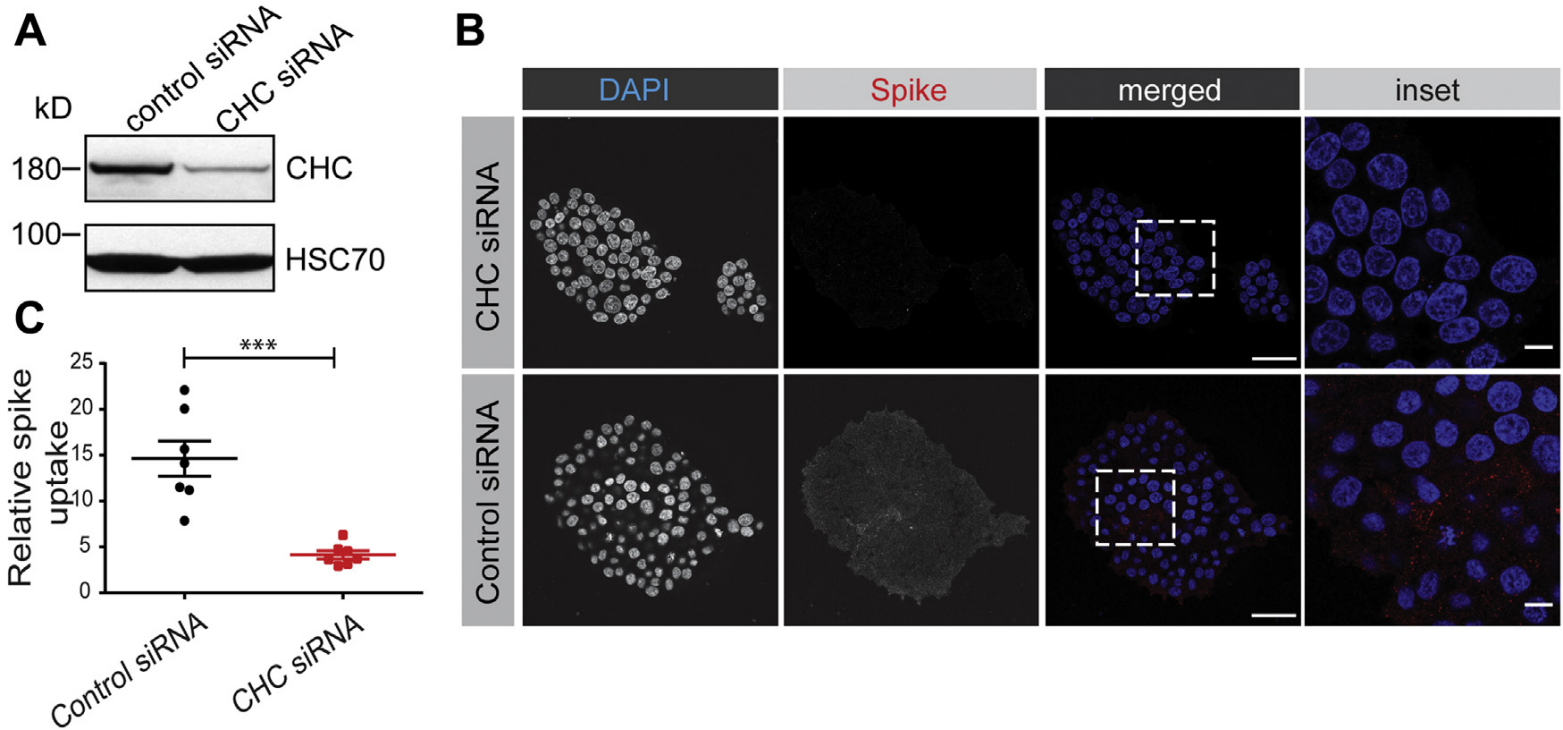
**SARS-CoV-2 spike protein endocytosis is reduced by CHC knockdown**. *A*, HEK-293T cells stably expressing ACE2 were transfected with control siRNA or with an siRNA established to selectively knockdown the expression of CHC. Cell lysates were prepared and immunoblotted with the indicated antibodies. *B*, HEK-293T cells stably expressing ACE2, transfected with a control siRNA or an siRNA driving CHC knockdown, were incubated with purified, His6-tagged spike protein for 30 min at 4 °C. The cells were then transferred to 37 °C for 30 min before being returned to ice. After acid wash, cells were fixed and stained with DAPI to reveal nuclei and with antibody recognizing the His6 epitope tag of the spike protein. Scale bars = 40 μm for the low-magnification images and 10 μm for the higher magnification *insets* on the right. *C*, quantification of experiments performed as in (*B*). n = 7 from three independent experiments, mean ± SEM; unpaired *t* test; ∗∗∗*p* < 0.001. ACE2, angiotensin-converting enzyme 2; CHC, clathrin heavy chain; SARS-CoV-2, severe acute respiratory syndrome coronavirus 2.

To best demonstrate a role for clathrin in spike protein uptake, we took a loss-of-function approach to inhibit clathrin-mediated endocytosis by knockdown of CHC with a previously defined siRNA pool (36, 37). Transfection of HEK-293T-ACE2 cells with the CHC siRNA led to an ∼70% decrease in CHC levels when compared with cells transfected with a control siRNA (Fig. 5*A*). Importantly, CHC knockdown led to a significant reduction of spike protein endocytosis (Fig. 5, *B* and *C*). Thus, the SARS-CoV-2 spike protein undergoes rapid internalization via clathrin-mediated endocytosis.

### SARS-CoV-2 spike protein undergoes rapid endocytosis in cell lines expressing endogenous levels of ACE2

**Figure 6:**
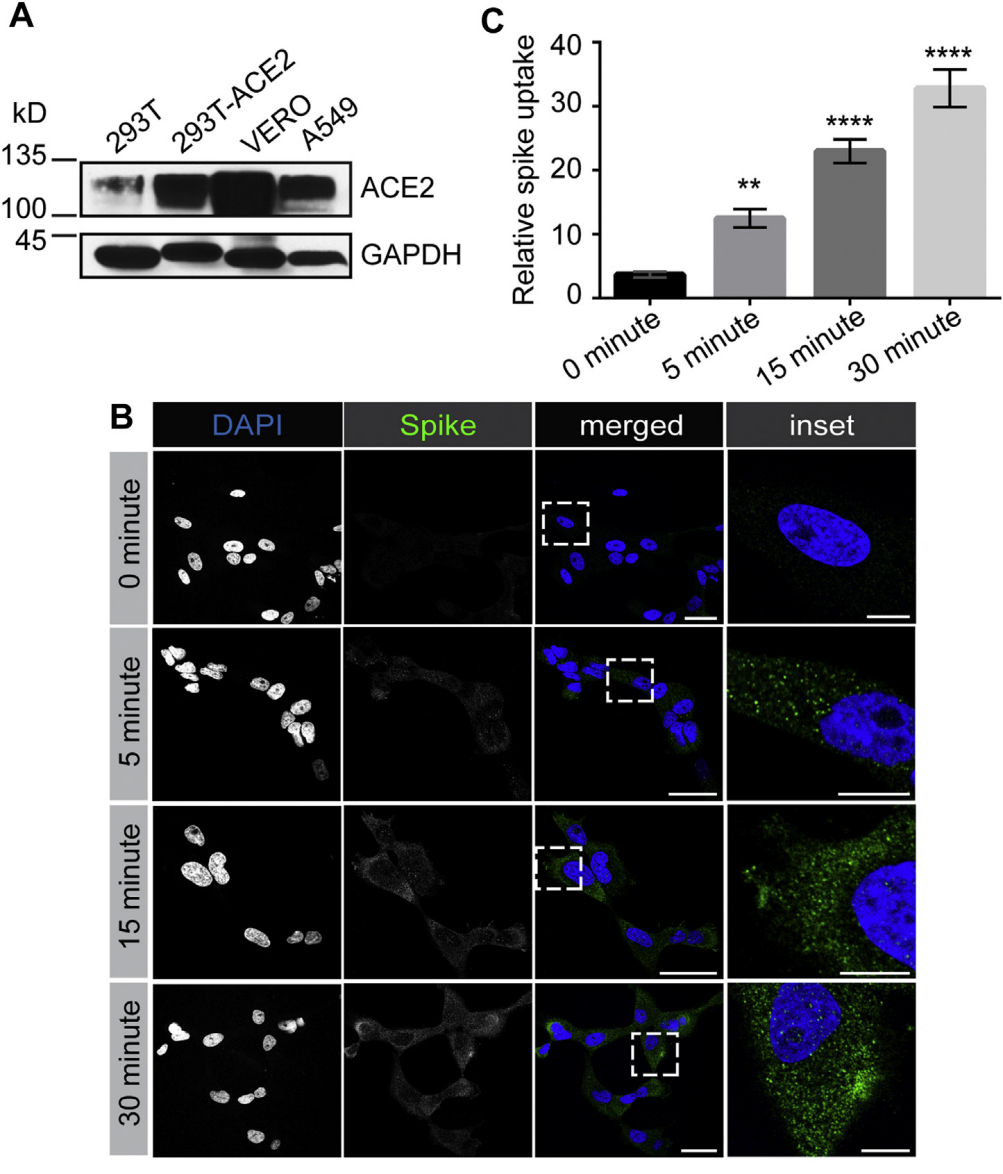
**SARS-CoV-2 spike protein is rapidly endocytosed in VERO cells**. *A*, lysates were made from HEK-293T cells, WT or expressing ACE2, VERO cells, and A549 cells and were immunoblotted with an antibody recognizing ACE2. *B*, VERO cells were incubated with purified, His6-tagged spike protein for 30 min at 4 °C. The cells were then transferred to 37 °C for the indicated time periods before being returned to ice. After acid wash, cells were fixed and stained with DAPI to reveal nuclei and with antibody selectively recognizing spike protein. Scale bars = 40 μm for the low-magnification images and 10 μm for the higher magnification *insets* on the right. *C*, quantification of experiments performed as in (*B*) from three independent experiments, mean ± SEM; one-way ANOVA; ∗∗∗∗*p* < 0.0001, ∗∗*p* < 0.01. ACE2, angiotensin-converting enzyme 2; SARS-CoV-2, severe acute respiratory syndrome coronavirus 2.

**Figure 7:**
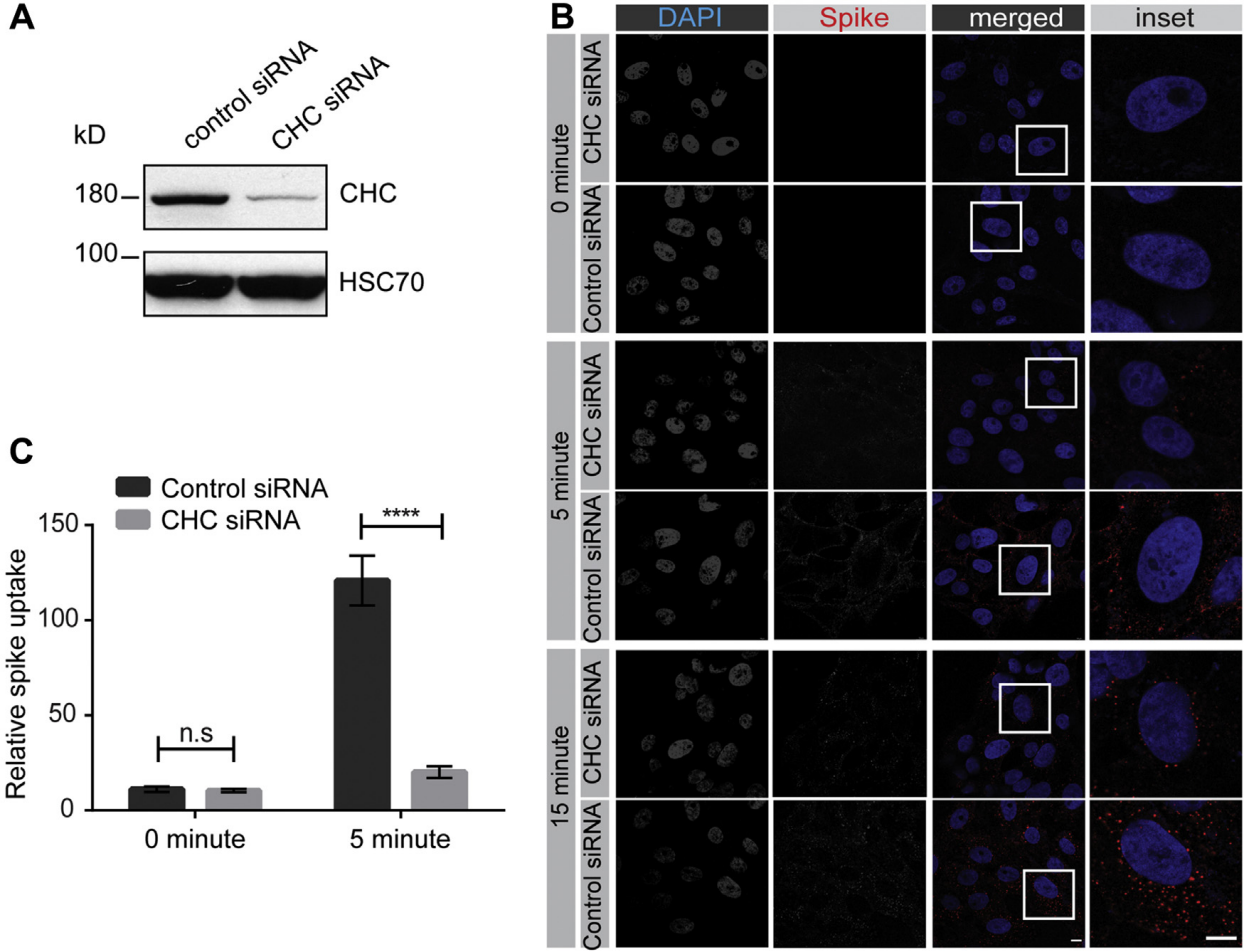
**SARS-CoV-2 spike protein endocytosis is reduced by CHC knockdown in VERO cells**. *A*, lysates were made from VERO cells transfected with control siRNA or with an siRNA established to selectively knockdown the expression of CHC. Cell lysates were prepared and immunoblotted with the indicated antibodies. *B*, VERO cells, treated as in (*A*), were incubated with purified spike protein for 30 min at 4 °C. The cells were then transferred to 37 °C for the indicated time periods before being returned to ice. After acid wash, cells were fixed and stained with DAPI to reveal nuclei and with antibody selectively recognizing spike protein. Scale bars = 40 μm for the low-magnification images and 10 μm for the higher magnification *insets* on the right. *C*, the graph showing quantification of experiments performed as in (*B*). n = 15 from 3 independent experiments, mean ± SEM; unpaired *t* test; ∗∗∗∗*p* < 0.0001, n.s, not significant. ACE2, angiotensin-converting enzyme 2; CHC, clathrin heavy chain; SARS-CoV-2, severe acute respiratory syndrome coronavirus 2.

We next sought to examine endocytosis of SARS-CoV-2 spike protein in cells expressing endogenous levels of the cellular receptor ACE2. We first tested VERO cells, a monkey kidney epithelial cell that is robustly infected by SARS-CoV-2 (9). VERO cells have high levels of ACE2 expression that exceeds even that of HEK-293T cells with overexpression of ACE2 (Fig 6*A*). SARS-CoV-2 spike protein added to VERO cells undergoes rapid endocytosis with the protein appearing in the cells as early as 5 min (Fig 6*B*), as determined using a spike protein antibody that recognizes the His6-tagged spike protein (Fig. S2). The amount of internalized protein increases slightly over the next 25 min, with the protein appearing as distinct punctae in the cells (Fig. 6*B*). These punctae colocalize in part with Rab5, a marker of early endosomes, indicating that a portion of the spike glycoprotein is delivered to early endosomes (Fig. S3, *A* and *B*). Knockdown of CHC in VERO cells (Fig. 7*A*) reduces spike glycoprotein internalization (Fig. 7, *B* and *C*), further supporting entry via clathrin-mediated endocytosis. We also tested for SARS-CoV-2 endocytosis in A549 cells that are widely used as a type II pulmonary epithelial cell model, and which are moderately infectible by the virus (9). A549 cells have ACE2 expression levels similar to the ACE2-expressing HEK-293T cell (Fig. 6*A*). These cells endocytose purified spike protein with the protein detectable in the cells at 5 min, and with increasing amounts of internalized protein up to 30 min (Fig. S4). As for the VERO cells, endocytosed spike protein appears as distinct punctae (Fig. S4). Thus, endocytosis of SARS-CoV-2 occurs in multiple cell types and is clathrin dependent.

### After endocytosis, SARS-CoV-2 spike protein has a distinct trafficking itinerary from Tf

**Figure 8:**
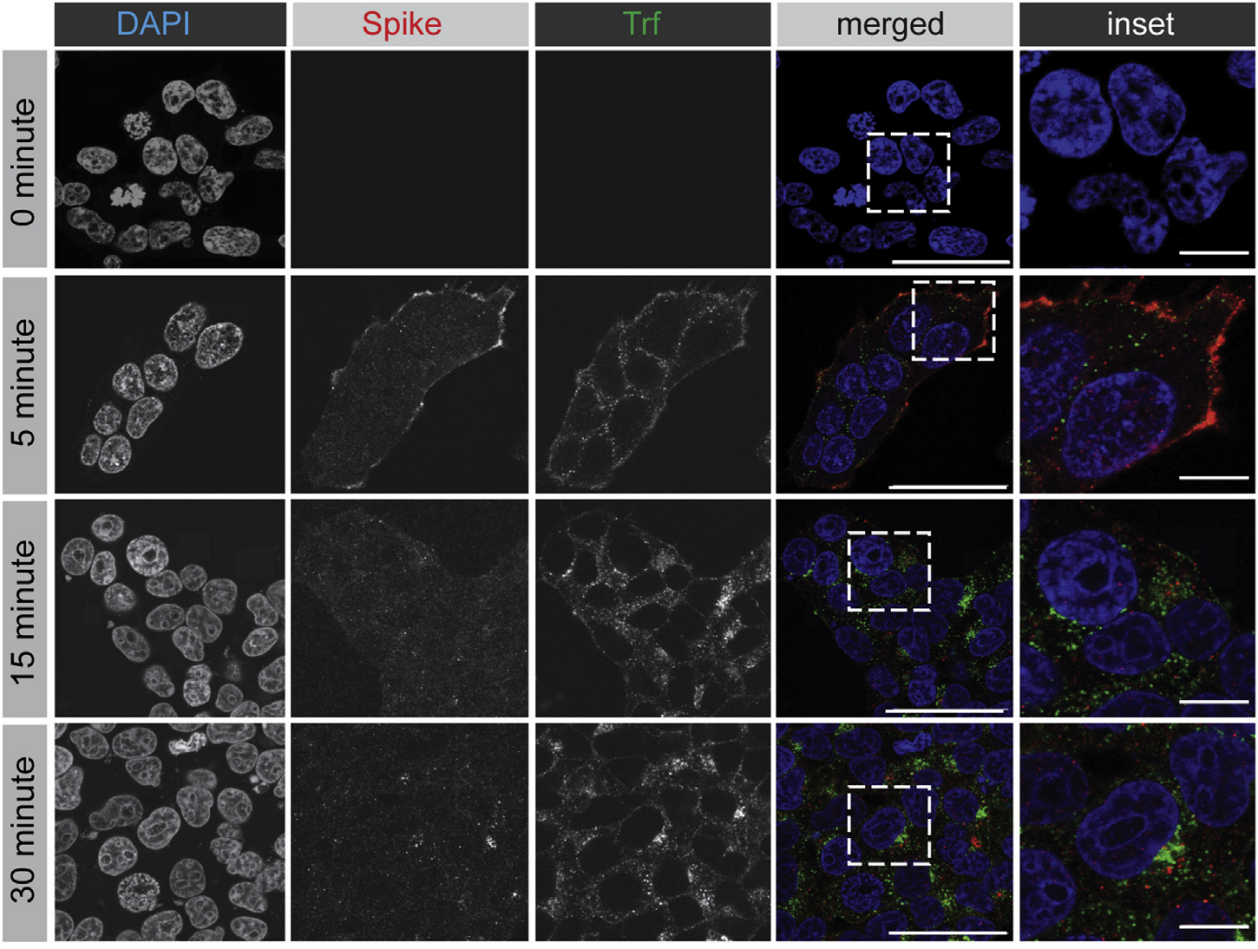
**SARS-CoV-2 spike protein follows a trafficking itinerary distinct from transferrin**. HEK-293T cells stably expressing ACE2 were incubated with purified, His6-tagged spike protein and Trf-Alexa647 for 30 min at 4 °C. The cells were then transferred to 37 °C for the indicated time periods before being returned to ice. After PBS wash, cells were fixed and stained with DAPI to reveal nuclei and with antibody selectively recognizing the His6 epitope tag of the spike protein. Scale bars = 40 μm for the low-magnification images and 10 μm for the higher magnification *insets* on the right. ACE2, angiotensin-converting enzyme 2; SARS-CoV-2, severe acute respiratory syndrome coronavirus 2.

Tf bound to the TfR is an established cargo of the recycling endosomal pathway. The Tf–TfR complex enters cells via clathrin-mediated endocytosis and rapidly transports to early endosomes. From there, the complex recycles directly back to the plasma membrane, a fast recycling route, or is transported to Rab11-positive recycling endosomes before returning to the cell surface, the slow recycling pathway (38). When spike protein and Tf are coincubated with HEK-293T cells stably expressing ACE2, both proteins are rapidly internalized and accumulate over 30 min (Fig. 8). However, there is little or no colocalization of the two cargo proteins once internalized (Fig. 8), indicating that they have different trafficking itineraries.

### Lentivirus pseudotyped with SARS-CoV-2 spike glycoprotein requires clathrin for infectability

**Figure 9:**
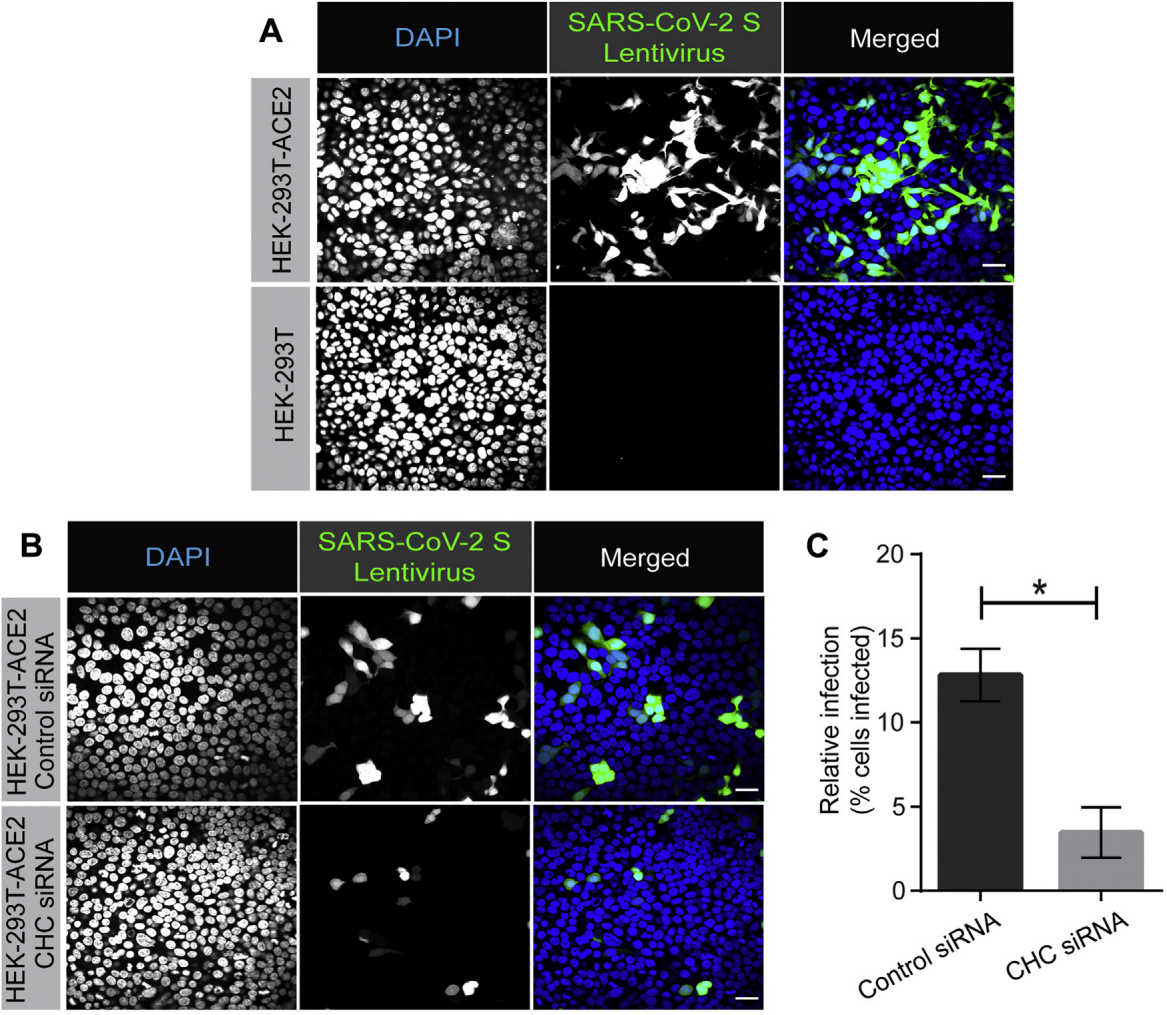
**Lentivirus pseudotyped with SARS-CoV-2 spike glycoprotein requires CHC for infectivity**. *A*, HEK-293T cells stably expressing ACE2 or HEK-293T cells were incubated with lentivirus pseudotyped with the SARS-CoV-2 spike glycoprotein for 12 h. Cells were then fixed and stained with DAPI to reveal nuclei. Cells were also imaged for GFP, driven from the pseudovirus. Scale bars = 40 μm. *B*, HEK-293T cells stably expressing ACE2 were transfected with control siRNA or with an siRNA established to selectively knockdown the expression of CHC. The cells were incubated with lentivirus pseudotyped with the SARS-CoV-2 spike glycoprotein for 12 h. Cells were then fixed and stained with DAPI to reveal nuclei. Cells were also imaged for GFP, driven from the pseudovirus. Scale bars = 40 μm. *C*, the graph showing quantification of SARS-CoV-2 pseudovirus infection from experiments as in (*B*). n = 3 from three independent experiments, mean ± SEM; unpaired *t* test, ∗*p* < 0.05. ACE2, angiotensin-converting enzyme 2; CHC, clathrin heavy chain; SARS-CoV-2, severe acute respiratory syndrome coronavirus 2.

We next sought to examine the role of clathrin-mediated endocytosis in viral infectivity. Viruses pseudotyped with the SARS-CoV-2 spike glycoprotein are a commonly used tool to examine the ability of SARS-CoV-2 to infect cells. Such pseudotyped viruses involve transfection of HEK-293T cells with a viral packaging construct, a viral transfer vector encoding luciferase or GFP, and a plasmid encoding the spike protein, and are based on murine leukemia virus (12), VSV (39), or lentivirus (30) among others. These pseudoviruses avoid the need for biocontainment 3 facilities. We have used a lentiviral system in which VSV-G was replaced with the SARS-CoV-2 spike glycoprotein (30). We first incubated the virus with HEK-293T cells, WT, or overexpressing ACE2, and infection was monitored by GFP expression. At 12 h of incubation, HEK-293T-ACE2 cells were infected, whereas little to no infection was seen with WT HEK-293T cells (Fig. 9*A*). We next repeated the experiment using HEK-293T-ACE2 cells transfected with a control siRNA or an siRNA driving knockdown of CHC (Fig. 5*A*). Importantly, CHC knockdown led to an ∼65% decrease in viral infectivity (Fig. 9, *B* and *C*). Taken together, our data indicate that SARS-CoV-2 uses its spike glycoprotein to engage ACE2, driving clathrin-mediated endocytosis of the virus/receptor complex, and that this process is required for viral infectivity.

## Discussion

Strategic entry mechanisms used by viruses to infect mammalian host cells are unique and highly variable across different viral families (40). Among the various routes used, the majority of viruses use endosomal pathways to efficiently deliver virion components into the cytoplasm for productive infection (41). Although the most commonly used endosomal pathway is clathrin-mediated endocytosis (used, *e.g.*, by Semliki forest virus and Adenoviruses 2 and 5), other forms of endocytosis are used (40, 41, 42). These include caveolae-mediated endocytosis (*e.g.*, Simian virus 40), lipid raft–mediated endocytic pathways (*e.g.*, Avian sarcoma leukosis virus), and macropinocytosis (*e.g.*, poxviruses) (40, 41, 42).

Endosomal entry mechanisms provide many advantages to the viruses by allowing them to efficiently spread while evading host immune surveillance. Most importantly, gaining access to the endocytic system helps viruses to prevent exposure of the viral capsid proteins to the host immune system at the level of plasma membrane. In addition, strategic use of endosomal pathway also ensures entry into cells with active membrane transport system as opposed to cells with no such means (*e.g.*, erythrocytes), which would prevent active propagation of the virus (40). Furthermore, the acidic milieu of endosomes helps the incoming viruses to elicit penetration into the host cytosol (40, 42). Finally, it has been recently demonstrated that egress of SARS-CoV-2 from cells uses a lysosomal pathway, further demonstrating the importance of the endosomal/lysosomal system to the virus life cycle (43).

The most closely related virus to SARS-CoV-2 is SARS-CoV. For SARS-CoV, endocytic entry has been suggested as the first step in infectivity with two mechanisms proposed, one clathrin-mediated endocytosis (27) with a second study indicating a clathrin-independent process (28). Here we used purified spike protein and lentivirus pseudotyped with SARS-CoV-2 spike glycoprotein to measure endocytosis and infectivity in HEK-293T cells expressing ACE2 and cell lines with endogenous ACE2 expression. We demonstrate that drugs, which are known inhibitors of clathrin-mediated endocytosis, block spike protein endocytosis and we further demonstrate that knockdown of CHC blocks both spike protein endocytosis and pseudovirus infectibility. We thus propose a viral infectivity model involving 3 key steps: (1) the virus uses its spike glycoprotein to bind to the plasma membrane of cells expressing ACE2. Notably, a recent paper questions that ACE2 is the cellular receptor for SARS-CoV-2 based on the observation that immunohistochemical analysis fails to detect ACE2 in cells and tissues that are infectible with virus (44). Our data do not support this observation; (2) the ACE2/virus complex undergoes rapid endocytosis with delivery to the lumen of the endosome; (3) fusion of the viral membrane with the lumen of the endosomal membrane allows viral RNA to enter the cytosol for infection. After entry, the viral particles appear in a pathway that is unique from the TfR. Because the TfR defines a recycling pathway, we propose the viral capsid proteins get targeted to late endosomes/lysosomes for degradation, even as the viral RNA is driving formation of new virus that egresses from lysosomes (43).

The discovery that clathrin-mediated endocytosis is an early step in viral infectivity will allow for a new emphasis on drug targets. Chloroquine (CQ) and hydroxychloroquine are widely used malaria drugs that have yielded mixed results for the treatment of COVID-19 (45). CQ is known for its efficacy in blocking the clathrin-mediated endocytosis of nanoparticles (46). CQ reduces the expression of the phosphatidylinositol-binding CHC assembly protein (46), and depletion of phosphatidylinositol-binding CHC assembly protein is known to block clathrin-mediated endocytosis (47). Hydroxychloroquine and CQ are in the class of aminoquinolines, which are hemozoin inhibitors, and were identified with other Food and Drug Administration–approved hemozoin inhibitors, including amodiaquine dihydrochloride dihydrate, amodiaquine hydrochloride, and mefloquine, in a screen testing for reduction of SARS-CoV-2 infectivity (48). Chlorpromazine, which is widely used to treat psychiatric disorders, blocks cellular entry of SARS-CoV and is known to disrupt clathrin-mediated endocytosis (27). This may explain why psychiatric patients treated with chlorpromazine have a lower incidence of COVID-19 (49). Thus, although vaccine development is clearly the lead mechanism to slow the pandemic, drugs that disrupt virus infectivity are likely to have a corollary role. Regardless of clinical implications, given the prevalence and severity of SARS-CoV-2 infection, and the likelihood that other viruses with pandemic implications will emerge, it behooves the scientific community to learn about all aspects of SARS-CoV-2 infectivity, including basic functions such as its precise mechanism of cellular entry. This study, which provides a clear demonstration that clathrin-mediated endocytosis is used by SARS-CoV-2 to enter cells, thus provides an important new piece of information on SARS-CoV-2 biology.

## Data availability

All data are contained within the manuscript.

## Conflict of interest

The authors declare that they have no conflicts of interest with the contents of this article.
