# Supplementary material for: SARS-CoV-2 infects cells after viral entry via clathrin-mediated endocytosis
Source: J Biol Chem. 2021 Jan 19;296:100306. doi: 10.1016/j.jbc.2021.100306 (PMC7816624; doi:10.1016/j.jbc.2021.100306)
Supplement: Supplementary file 1 — Figures S1 to S4 [file mmc1.pdf]

Supplemental information

SARS-CoV-2 infects cells following viral entry via clathrin-mediated endocytosis

Armin Bayati*, Rahul Kumar*, Vincent Francis, and Peter S. McPherson**

* These two authors contributed equally to this study

Department of Neurology and Neurosurgery, Montreal Neurological Institute, McGill University, Montreal, QC, Canada

**To whom correspondence should be addressed: Department of Neurology and Neurosurgery Montreal Neurological Institute, McGill University, 3801 University Street Montreal, QC H3A 2B4, Canada, phone: (514) 398-7355, Email: peter.mcpherson@mcgill.ca

Running title: endocytosis of SARS-CoV-2

Key words: clathrin, COVID-19, dynamin, endocytosis, infection, SARS-CoV-2, virus entry

Supplemental figure legends

Supplemental Figure 1. Dynasore and pitstop do not disrupt dextran uptake. (A) HEK-293T cells stably expressing ACE2 were incubated with dextran or Tf for 30 min at 4°C either in the presence of DMSO, dynasore in DMSO, Pitstop or a Pitstop negative control. The cells were then transferred to 37°C for 30 min before being returned to ice. Following acid wash, cells were fixed and stained with DAPI to reveal nuclei, antibody recognizing the His6 epitope tag of the spike protein, and alexa-647 labelled Tf. Scale bars = 50 μm. **(B)** Quantification of experiment as in **A** from three independent experiments, mean ± SEM; unpaired t-test; *, $p < 0.05$, ****, $p < 0.0001$.

Supplemental Figure 2. The antibody against SARS-CoV-2 spike protein specifically recognizes internalized protein. HEK-293T cells stably expressing ACE2 were incubated with purified, His6-tagged spike protein for 30 min at 4°C. The cells were then transferred to 37°C for the indicated time periods before being returned to ice. Following acid wash, cells were fixed and stained with DAPI to reveal nuclei and with antibody recognizing the His6 epitope tag of the spike protein and with antibody directed against the spike protein. Scale bars = 40 μm for the low mag images and 10 m for the higher mag insets on the right.

Supplemental Figure 3. SARS-CoV-2 spike protein partially co-localizes with Rab5 following endocytosis. VERO cells were incubated with purified spike protein for 30 min at 4°C. The cells were then transferred to 37°C for the indicated time periods before being returned to ice. Following acid wash, cells were fixed and stained with antibody recognizing Rab5 and the spike protein. Scale bars = 10 μm. Quantification of Pearson's correlation coefficient of Rab5 and spike.

Supplemental Figure 4. SARS-CoV-2 spike protein is rapidly endocytosed in A549 cells. A549 cells were incubated with purified, His6-tagged spike protein for 30 min at 4°C. The cells were then transferred to 37°C for the indicated time periods before being returned to ice. Following acid wash, cells were fixed and stained with DAPI to reveal nuclei and with antibody selectively recognizing spike protein. Scale bars = 40 μm for the low mag images and 10 μm for the higher mag insets on the right.

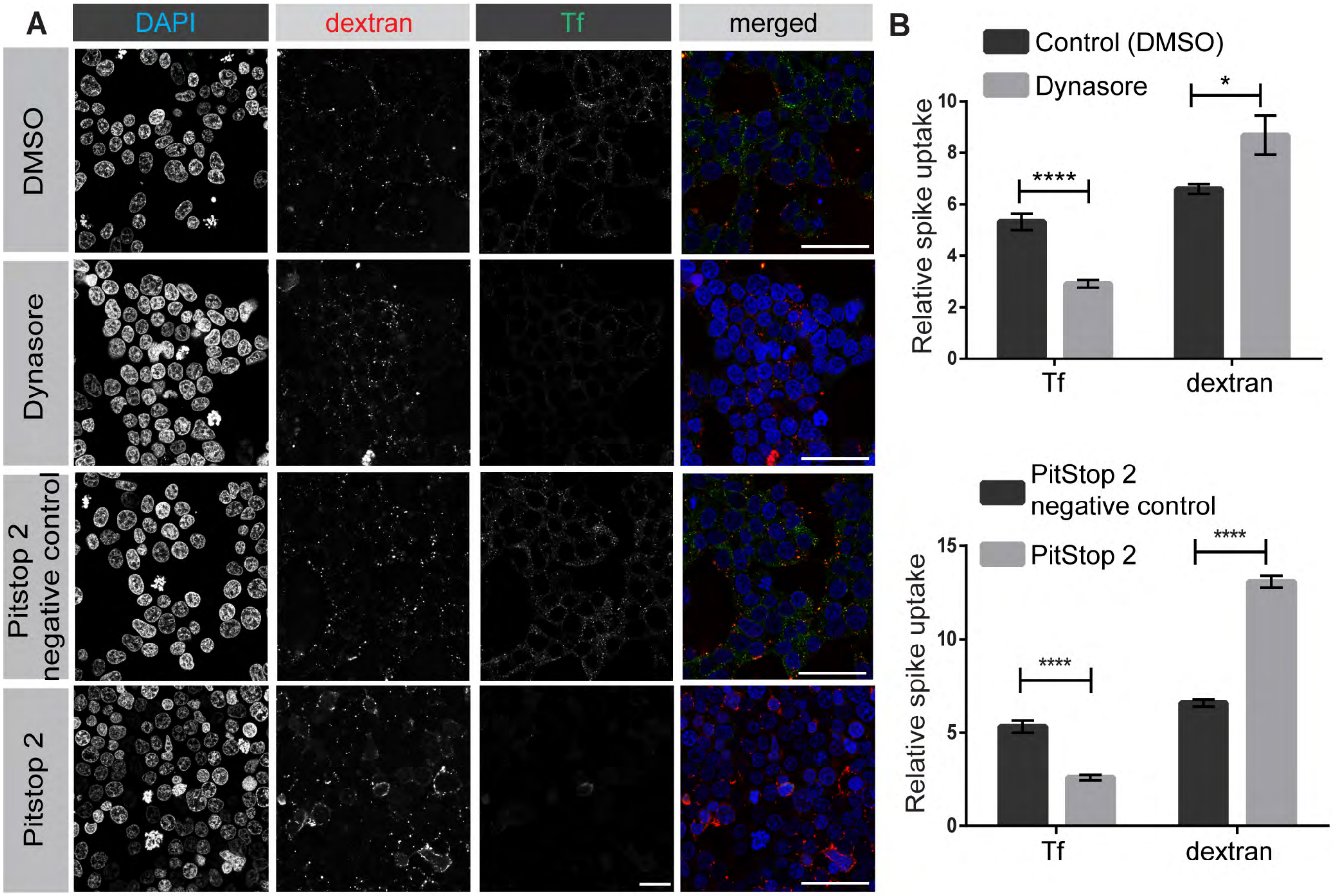

Bayati et al., Supp Fig.1

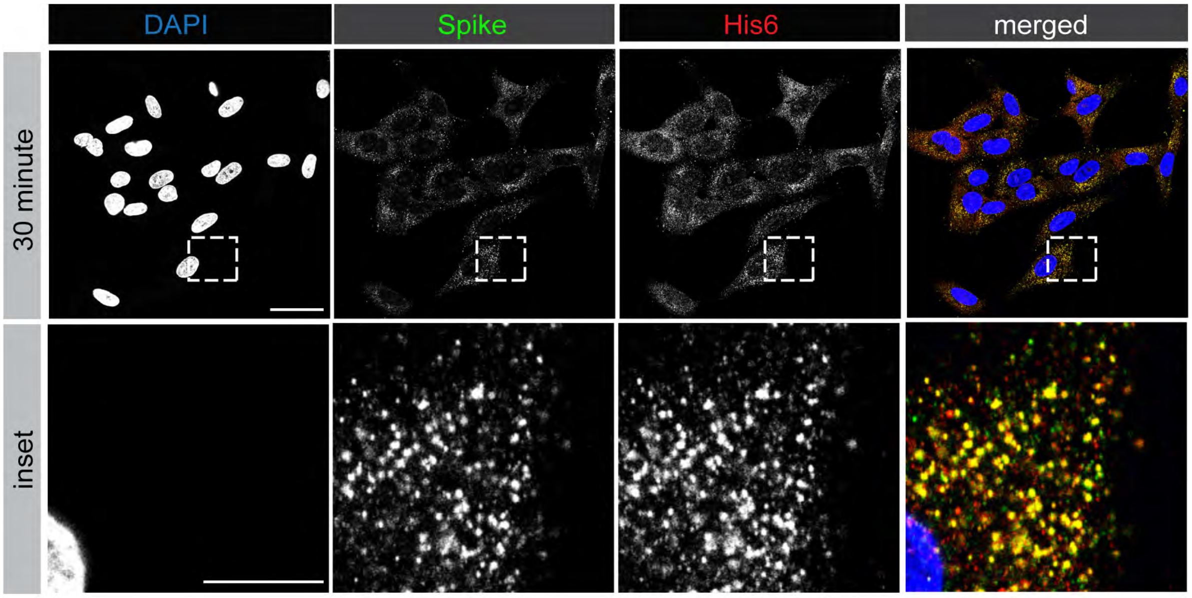

Bayati et al., Supp Fig.2

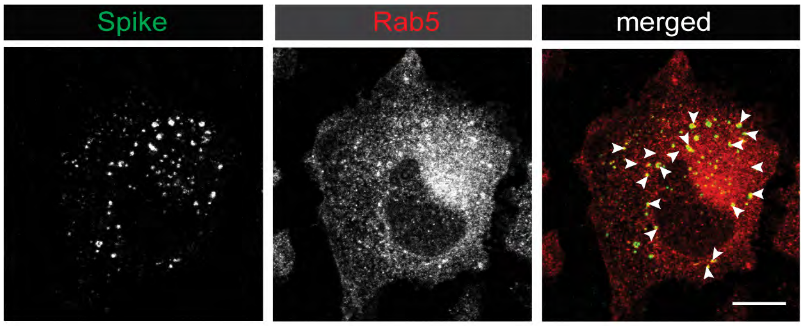

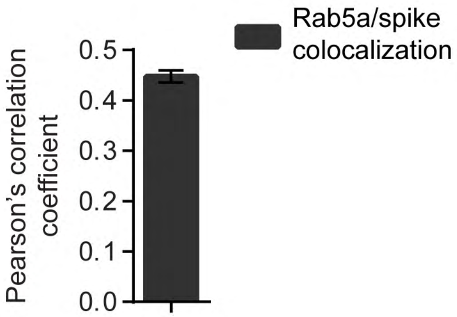

Bayati et al., Supp Fig.3

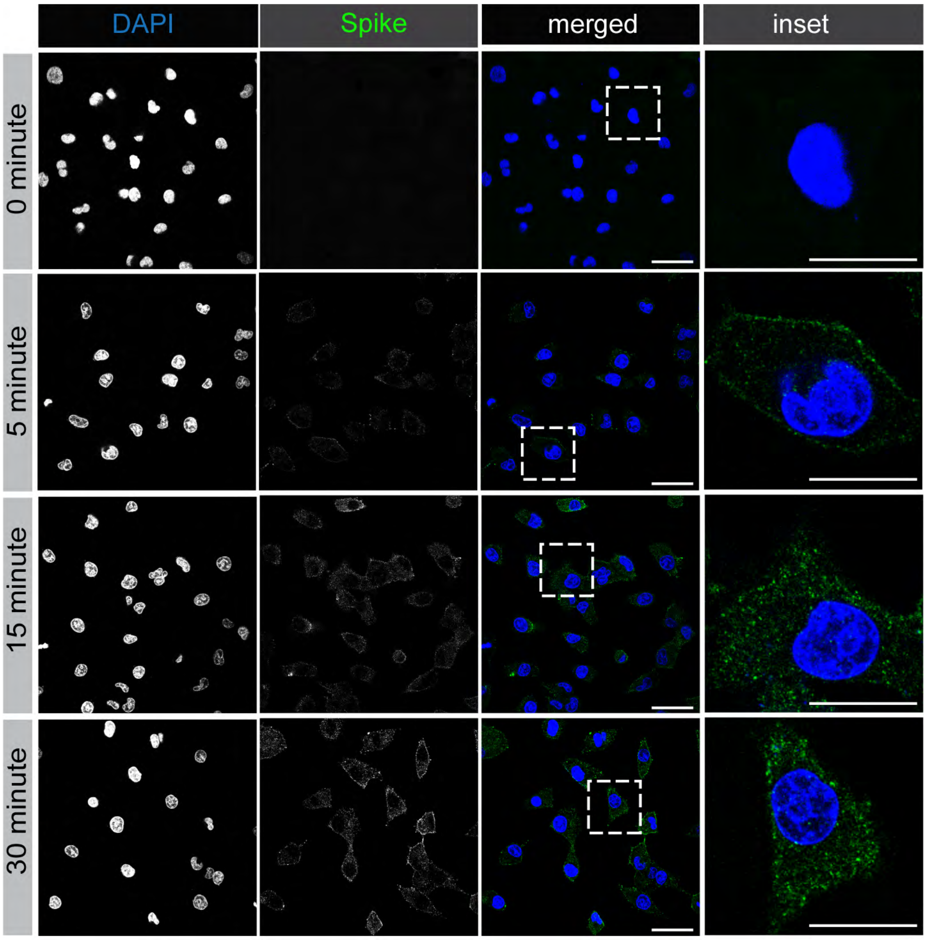

Bayati et al., Supp Fig.4
